# O-Vanillin Attenuates the TLR2 Mediated Tumor-Promoting Phenotype of Microglia

**DOI:** 10.3390/ijms21082959

**Published:** 2020-04-22

**Authors:** Paul Triller, Julia Bachorz, Michael Synowitz, Helmut Kettenmann, Darko Markovic

**Affiliations:** 1 Max Delbrueck Center for Molecular Medicine in the Helmholtz Association, Robert-Rössle-Str 10, 13125 Berlin, Germany; paul.triller@charite.de (P.T.); julia.bachorz@charite.de (J.B.); kettenmann@mdc-berlin.de (H.K.); 2 Neurosurgery Department, Schleswig Holstein University Clinic, Arnold-Heller-Straße 3, 24105 Kiel, Germany; Michael.Synowitz@nch.uni-kiel.de; 3 Neurosurgery Department, Helios Clinics Berlin-Buch, Schwanebecker Chaussee 50, 13125 Berlin, Germany

**Keywords:** microglia, glioma, therapy, tumor microenvironment, TLR2

## Abstract

Malignant gliomas are primary brain tumors with poor prognoses. These tumors are infiltrated by brain intrinsic microglia and peripheral monocytes which promote glioma cell invasion. In our previous studies, we discovered that the activation of Toll-like receptor 2 (TLR2) on microglia/brain macrophages converts them into a protumorigenic phenotype through the induction of matrix metalloproteinases (MMP) 9 and 14. In the present study, we used in vitro and in situ microglia-glioma interaction experimental models to test the impact of a novel inhibitor of TLR 2, ortho vanillin (O-Vanillin) to block TLR2 mediated microglia protumorigenic phenotype. We demonstrate that O-Vanillin inhibits the TLR2 mediated upregulation of *MMP 9*, *MMP 14*, *IL* 6 and *iNOS* expression. Similarly, the glioma supernatant induced *MMP 9* and *MMP 14* expression in murine and human microglia is abrogated by O-Vanillin treatment. O-Vanillin is not toxic for microglia, astrocytes or oligodendrocytes. Glioma growth in murine brain slice cultures is significantly reduced after treatment with O-Vanillin, and this reduced glioma growth depends on the presence of microglia. In addition, we also found that O-Vanillin inhibited the glioma induced proliferation of murine primary microglia. In summary, O-Vanillin attenuates the pro-tumorigenic phenotype of microglia/brain macrophages and thus qualifies as a candidate for glioma therapy.

## 1. Introduction

Primary malignant brain tumors remain a devastating tumor entity characterized by poor prognoses despite extensive research and gains in knowledge concerning its genesis and progression [1]. Microglia, the innate immune cells of the brain and infiltrating monocytes/macrophages play a pivotal role in glioma promotion and growth and contribute to the tumor tissue by up to 30% of the cells [2]. Glioma induces the polarization of glioma associated microglia/macrophages (GAMs) into a glioma-supporting phenotype through complex interactions involving several signaling mechanisms [3]. One major mechanism that contributes to glioma invasiveness is the degradation of the surrounding extracellular matrix by matrix metalloproteases (MMPs). MMPs are a group of enzymes that proteolytically cleave diverse proteoglycans, collagens and gelatin [4]. We discovered that microglial cells express MMP14 as a membrane bound protein which serves to activate glioma released MMP2. While microglia in the healthy brain do not express MMP14, it is upregulated in glioma associated GAMs. This upregulation is induced by versican released from glioma cells which signals via toll like receptor 2 (TLR2). This interaction between glioma and GAMs is an important contributor to microglia/macrophage-induced glioma invasiveness und progression [5,6]. TLRs are membrane-bound receptors that recognize a broad spectrum of pathogen-associated molecular patterns. In addition, TLRs can be activated by endogenous ligands such as versican and tenascin C [7]. In the glioma context, TLR4 and in particular TLR2 are mediators of glioma-GAM interactions. The expression of TLR2 and MMPs in glioma tissue was found to be inversely correlated with patients’ median survival according to TCGA analysis. In our previous study, we demonstrated that the downregulation of TLR2 in microglia by minocycline treatment correlates with the downregulation of MMP14 and increased survival of brain tumor bearing mice [5]. Recently, Mistry et al. [8] identified a loop within the intracellular adapter domain of TLR2 that can be blocked by O-Vanillin, and thus identified O-Vanillin as an TLR2 inhibitor. O-Vanillin is an isomer of the well-known food supplement vanillin and was studied in different malignancies including colon, ovarian and lung cancer and melanoma [9,10,11]. So far, the use of O-Vanillin as a potential drug in gliomas has not yet been explored.

In the present study, we tested whether O-Vanillin can be used to inhibit glioma-induced TLR2 signaling in murine microglia. O-Vanillin was able to attenuate the glioma-induced increase in *mmp14* and *mmp9* in cultured microglia as well as in microglia/macrophages isolated from human glioma samples. Moreover, O-Vanillin also attenuated the growth of gliomas injected into murine brain slice cultures and this effect was dependent on the presence of microglia.

## 2. Results

### 2.1. Vanillin Blocks TLR2 Mediated Signaling in Microglia

**Figure 1:**
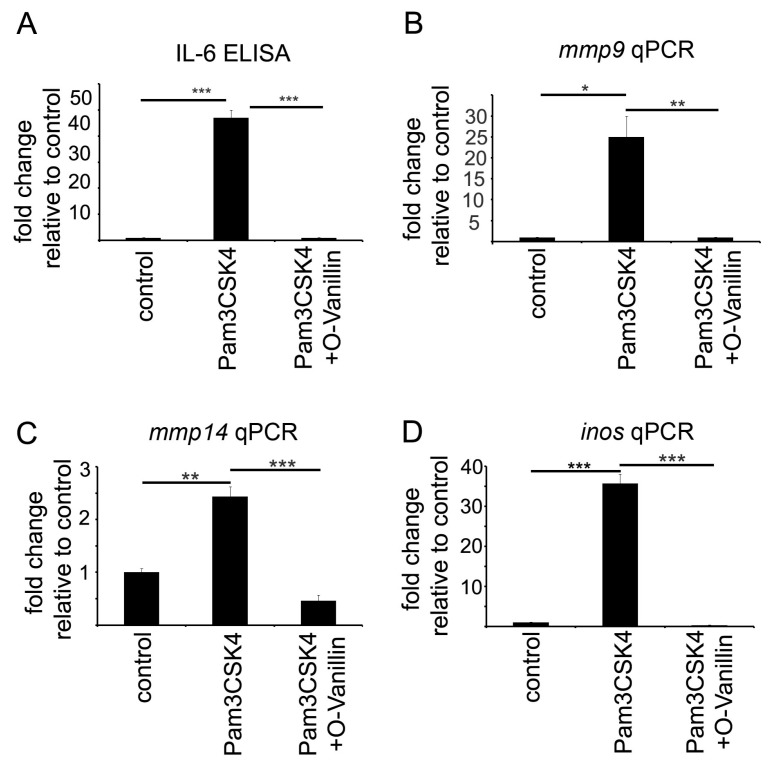
O-Vanillin abrogates the Pam3CSK4 induced TLR2 stimulation. We stimulated microglial cells with the TLR2 agonists Pam3CSK4 (1 µg/mL) and with Pam3CSK4 combined with 100 µM O-Vanillin compared to control in DMEM. We determined relative increase in IL6 by ELISA (**A**) and *mmp9* (**B**), *mmp14* (**C**) and *inos* by qPCR (**D**) 6 h after stimulation. The panel shows means and SEM from three independent experiments. Significance was defined at *p* values < 0.05 (*), <0.01 (**) and <0.001 (***).

To analyze O-Vanillin’s potential as inhibitor of TLR2 signaling in microglia, we stimulated primary cultured microglia cells with the TLR2 agonists Pam3CSK4 (1 µg/mL) for 6 h and measured the release of IL-6 by ELISA (Figure 1A) and the increased RNA expression of *mmp9* (Figure 1B), *mmp14* (Figure 1C) and *inos* (Figure 1D) by PCR. Pam3CSK4 significantly increased the release of IL-6 by 46 times and the RNA expression of *mmp9* by 25 times, of *mmp14* by 2.5 times and of *inos* by 35 times. Co-incubation with 100 µM O-Vanillin reduced the IL-6 levels and the increase in *mmp9*, *mmp14* and *inos* RNA expression to control levels (Figure 1), indicating that O-Vanillin is a highly efficient inhibitor of TLR2 in murine microglia.

### 2.2. Vanillin is Not Cytotoxic to Glial Cells and Only at High Doses to Glioma Cells

**Figure 2:**
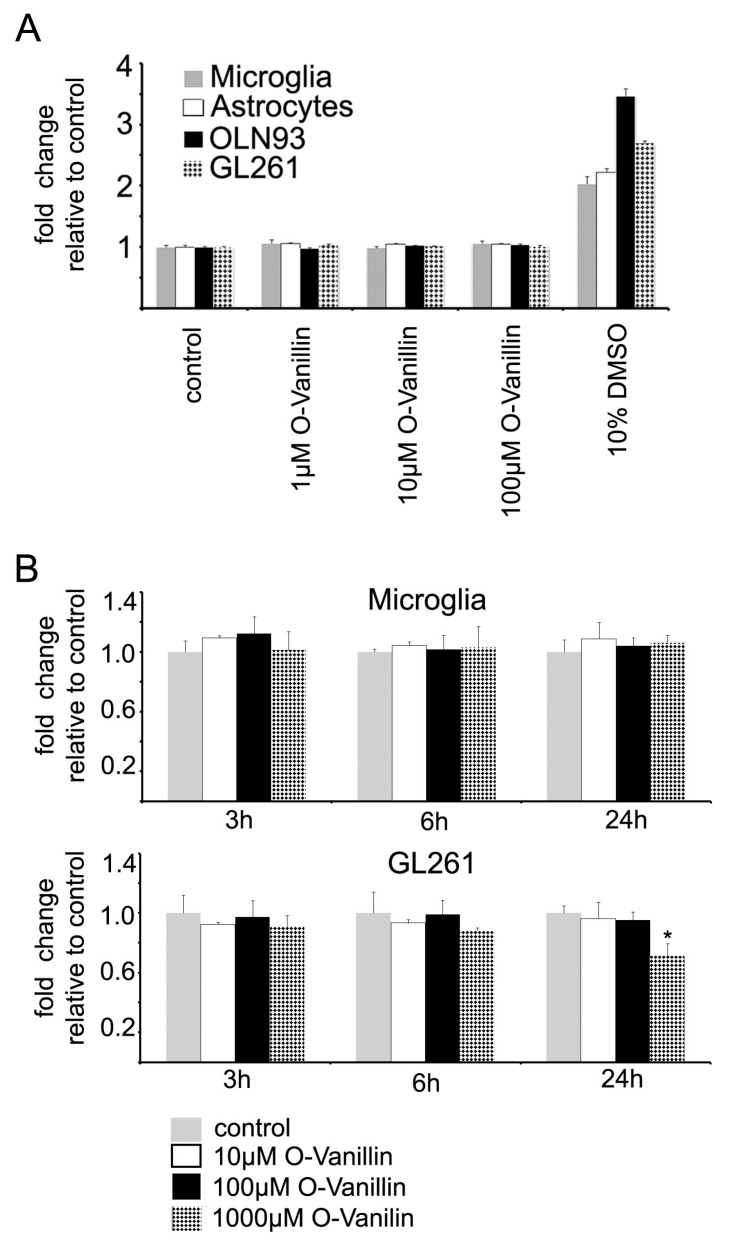
Vanillin is not cytotoxic to glial cells and only at high doses to glioma cells (**A**) PI incorporation in microglial cells, astrocytes, OLN93 cells and GL261 cells is not affected by treatment with O-Vanillin. Microglia, astrocytes, oligodendrocytic cell line OLN93 and GL261 were treated with O-Vanillin at 1, 10, 100 µM for 24 h. Subsequently, we determined the PI incorporation by measuring red fluorescence relative to the control in DMEM only. The incorporation of PI did not change due to O-Vanillin treatment. Application of 10% DMSO served as a positive control. (**B**) O-Vanillin reduces cell metabolism in glioma GL261 cells but not in microglial cells. Primary murine microglia cells were treated with 10, 100, 1000 µM O-Vanillin at the concentrations for 3, 6 and 24 h. The metabolic activity of the cells was determined with the Alamar Blue assay. Cells incubated in cell culture medium only served as control. No significant alteration in cell metabolism were observed at any condition. In a similar approach, the metabolic activity of GL261 glioma cells was analyzed. A significant reduction in cell metabolism in comparison to the control group was observed after stimulation with 1000 µM O-Vanillin after 24 h of incubation (**B**). The panel shows means and SEM from three independent experiments. Significance was defined at *p* value < 0.05 (*).

To test the potential cytotoxicity of O-Vanillin, we evaluated the cell viability of cultured murine microglia and astrocytes, the oligodendrocyte cell line OLN 93 and GL261 glioma cells by analyzing the propidium iodide (PI) incorporation as marker for cell death in a plate reader. Cells were incubated for 24 h with 1, 10 and 100 µM O-Vanillin. We did not observe more than 3% differences in the PI fluorescence at all O-Vanillin concentrations in all the four cell types (Figure 2A). As a positive control, we incubated cells with 10% DMSO, which resulted in a more than 100% increase in the number of PI fluorescent cells (Figure 2A). Increasing the exposition time of 100 µM O-Vanillin to 48 h did also not affect the number of PI fluorescent microglial cells. To validate these results, we also analyzed the PI incorporation in microglial cells after 24h treatment with 1, 10 and 100 µM O-Vanillin via FACS. Similar to PI fluorescence measured in the plate reader, there was no difference in PI positive microglial cells when compared to control (Figure S1). As another assay for cell viability, we used the Alamar Blue assay which indicates metabolic activity. After incubation of microglial and GL261 cells with 10 and 100 µM O-Vanillin, we measured no significant difference in the Alamar Blue assay (Figure 2B). Incubation of microglial cells with 1000 µM O-Vanillin did not have an effect. Only GL261 cells incubated for 24 h with 1000 µM O-Vanillin showed decreased metabolic activity (Figure 2B).

### 2.3. O-Vanillin Attenuates the GCM Stimulated Expression of MMP9 and MMP14

**Figure 3:**
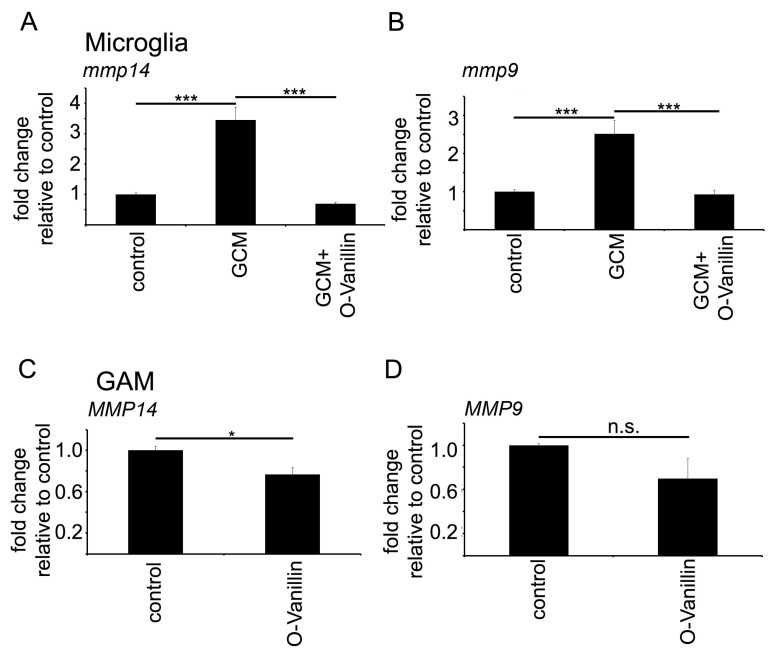
O-Vanillin significantly reduces the glioma induced increase of the TLR2 downstream targets *mmp9* and *mmp14* on RNA level in murine primary microglial cultures (**A**,**B**) and CD11b+ human GAM (**C**,**D**). GCM induced a significant increase in the RNA expression of *mmp14* (**A**) and *mmp9* (**B**) in murine primary microglia cells with GCM treatment and could inhibit this effect by treating the cells with 100 µM O-Vanillin for 6 h. (**C**) and (**D**) we treated GAM from human glioma samples for 12 h with O-Vanillin. The expression of *MMP14* (**C**) was significantly reduced by 26%. In D) the reduction in *MMP9* RNA levels after O-Vanillin treatment was not significant. The panel shows means and SEM from three independent experiments. Significance was defined at *p* values < 0.05 (*), <0.001 (***) and n.s. not significant.

We have previously shown that GCM triggers an increase in *mmp9* and *mmp14* expression in cultured microglia which is mediated by TLR2 signaling [12,13]. We confirmed the increase in *mmp9* and *mmp14* expression after a 6 h stimulation of cultured microglia with GCM obtained from the supernatant of GL261 cells. *mmp9* expression was increased to 2.6 and *mmp14* to 3.2-fold as determined with PCR. Co-incubating the microglial cells with GCM and 100 µM O-Vanillin completely abolished the stimulating effect of GCM on *mmp9* and *mmp14* expression (Figure 3A,B).

We further confirmed these findings in human glioma associated CD11b+ cells (i.e., glioma associated microglia and macrophages-GAM). We isolated CD11b+ cells from human glioma samples and cultivated them for 12 h. The cells were incubated with 100 µM O-Vanillin. The treatment with O-Vanillin significantly reduced the RNA expression of *MMP14* by 26% in GAM (Figure 3C). The reduction in *MMP9* was not significant (Figure 3D).

### 2.4. O-Vanillin Reduces Glioma Growth in Organotypic Brain Slices

**Figure 4:**
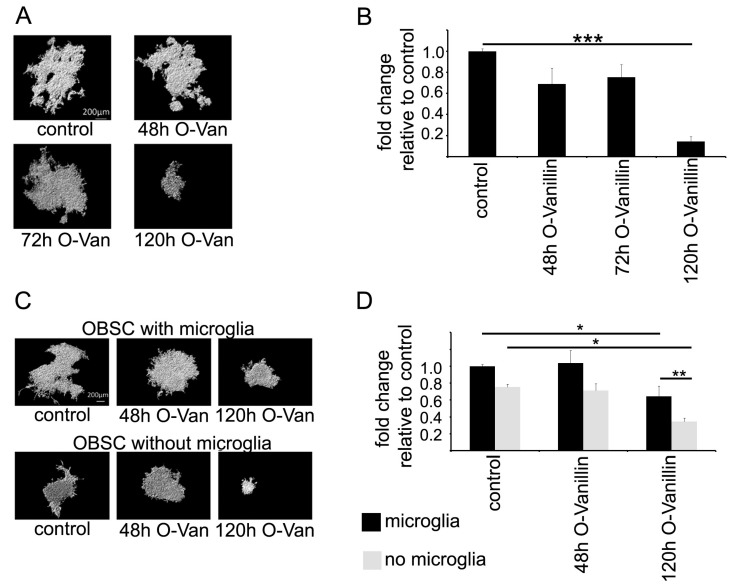
O-Vanillin reduced tumor volume in organotypic brain slices (OBSC) and the reduction is further enhanced after depletion of microglia. (**A**) OBSC were treated with 100 µM O-Vanillin for 48 h, 72 or 120 h after inoculation with red fluorescent mCherry GL261 glioma cell line into the slice. Examples for 3D reconstruction of tumor spread in each group are shown. (**B**) Mean tumor volume for all experiments are shown relative to the control. We observed a significant reduction in tumor volume by 85% in comparison to the control group after 120 h O-Vanillin treatment. Both 72 and 48 h O-Vanillin treatment did not significantly reduce tumor volume. (**C**) To investigate whether reduction in tumor volume after O-Vanillin treatment is microglia dependent, we depleted microglial cells using clodronate liposomes and treated OBSC with O-Vanillin for 48 and 120 h after inoculation of fluorescent labeled GL261 murine glioma cells. Examples for 3D reconstruction of tumor spread in each group are shown. (**D**) Mean tumor volume for all experiments are shown relative to the control. We observed a significant reduction in tumor volume by 24% in OBSC’s depleted of microglia, reduction in tumor volume by 35% after treatment with O-Vanillin without microglia depletion as well as reduction in tumor volume by 65% after both depletion of microglia and O-Vanillin treatment for 120 h in comparison to untreated control group. The reduction in tumor volume by 30% after both microglia depletion and O-Vanillin treatment for 120 h in comparison to the group without microglia depletion but after treatment with O-Vanillin for 120 h was significant. The panel shows means and SEM from three independent experiments. Significance was defined at *p* values < 0.05 (*), <0.01 (**) and <0.001 (***).

Organotypic brain slices were injected with GL261 glioma cells as an experimental model to quantify glioma growth. We injected GL261 cells expressing red fluorescent protein mCherry into organotypic brain slices obtained from mouse cortex 24 h after slice preparation and quantified glioma growth after 120 h by measuring the volume of the fluorescent signal after 3D reconstruction with the Imaris software. The treatment with 100 µM O-Vanillin during the entire period of 120 h significantly reduced the tumor volume by 85% compared to an untreated control. When the O-Vanillin treatment was delayed, by either 48 or 72 h, there was no significant reduction in the fluorescent signal (Figure 4A,B).

We have previously shown that microglia promote glioma growth in the organotypic brain slice model [14]; therefore, we analyzed the effect of O-Vanillin in brain slices depleted of microglia. By treating the organotypic slices for 24 h with clodronate-filled liposomes, we depleted microglia as previously described [14]. After a resting period of 72 h, the slices were inoculated with mCherry Gl261 cells and analyzed after 120 h. As reported previously, the fluorescence volume was reduced by 24% when comparing untreated slices without microglia to slices with microglia. The 120 h treatment with 100 µM O-Vanillin reduced glioma volume by 38% in microglia containing slices. The tumor volume was reduced by 65% in OBSC without microglia 120 h after 100 µM O-Vanillin treatment. Again, the 48 h treatment with 100 µM O-Vanillin did not significantly alter tumor volumes in control and experimental groups (Figure 4C,D). Please note that the Figure 4B,D do not represent the same kind of experiments. In Figure 4B, the slices were inoculated 24 h after preparation with mCherry Gl261 cells, and in Figure 4D, 96 h after preparation, due to the microglia depletion protocol [14].

### 2.5. O-Vanillin Reduces the Glioma-Induced Proliferation of Cultured Microglia

**Figure 5:**
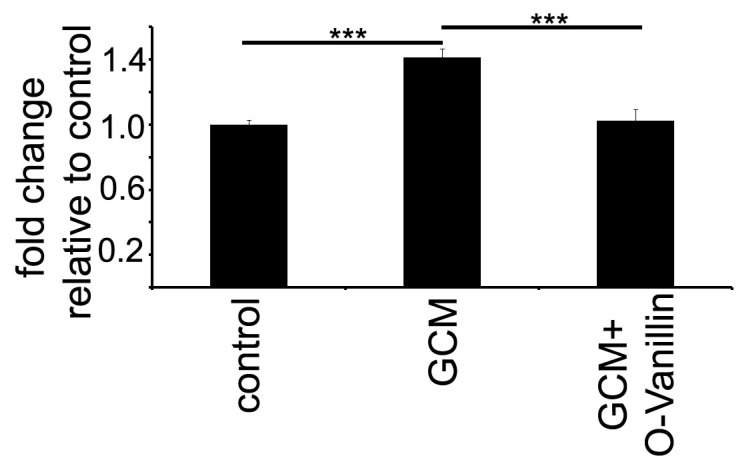
O-Vanillin significantly reduced glioma induced microglia proliferation.The proliferation rate was determined by BrdU incubation for 24 h and is shown relative to control. The stimulation with GCM significantly increased the proliferation by 41%. Co-application of GCM and 100 µM O-Vanillin normalized the proliferation to control level. The panel shows means and SEM from three independent experiments. Significance was defined at *p* value <0.001 (***).

We stimulated murine primary cultured microglia with GCM and measured the proliferation rate by BrdU incorporation. Application of GCM increased the proliferation rate of microglial cells by 41% after 24 h (Figure 5). Co-application of 100 µM O-Vanillin inhibited the effect of GCM on microglia proliferation (Figure 5).

## 3. Discussion

Glioma cells polarize GAMs into a tumor promoting phenotype and these cells acquire an expression profile which is distinct from M1 or M2 profile [15]. Their tumor promoting activity is mediated by an increased production and activation of matrix metalloproteinases, in particular MMP9 and MMP14 [12,13]. Thus, glioma cells exploit GAMs to promote glioma invasiveness, immune evasion and survival [3]. TLRs induce the glioma-associated phenotype of GAMs. We have shown that the endogenous ligand versican activates TLR2 in GAMs and is an important pathway for glioma-GAM communication [12]. The TLR2 pathway has been established as a tumor promoting component in different malignancies like ovarian carcinomas, melanomas, or gliomas [9,16]. The expression of TLR2 in glioma samples, which consists of up to 30% of microglia/macrophages, is inversely correlated with patients’ overall survival [6,17]. We also found a negative correlation between *MMP14* and *MMP9* expression on overall survival [18]. Thus, TLR2 has emerged as a novel target for impairing glioma growth and we could demonstrate in a mouse glioma model that interfering with TLR2 signaling by using TLR2—specific antibodies attenuated glioma growth [6].

Mistry et al. identified O-Vanillin using computer-aided structure analysis as potential inhibitor of TLR2 signaling by blocking its heterodimerization. They were able to inhibit the TLR2-mediated increase in *IL8* RNA in peripheral macrophages using O-Vanillin. We adapted these finding to the context of GAMs. We showed that O-Vanillin is a specific TLR2 inhibitor in primary microglia (Figure 1). We found that the TLR2 agonist Pam3CSK4 induced an increased *mmp9*, *mmp14*, and *inos* expression [19,20,21]. A quantity of 100 µM O-Vanillin was able to inhibit this induction. Moreover, the increase in *MMP9* and *MMP14* expression by GCM was also blocked by O-Vanillin. We therefore conclude that O-Vanillin works as an TLR2 inhibitor in microglia.

To test the impact of O-Vanillin on glioma growth, we used an organotypic slice model in which we could quantify glioma growth. Treatment of the slices with 100 µM O-Vanillin led to a significant reduction in tumor volume by 85%. We also tested whether the impact of O-Vanillin was mediated by microglia by depleting these cells from the slices. As previously described, there was a reduction in glioma growth through the depletion of microglia, but treatment with O-Vanillin led even to a further decrease in glioma growth. This can be explained by our observation that O-Vanillin directly inhibited glioma growth. As a further mechanism of O-Vanillin action, we observed an inhibitory effect on microglia proliferation induced by GCM. It is, however, not yet fully explored to what extent and by which mechanism glioma induce the proliferation of microglia. In contrast to our observation in mice, conditioned medium from the rat glioma cell line C6 did not alter rat microglial proliferation [21]. Li and Graeber (2012) speculate that gliomas secrete monocyte colony stimulating factor (M-CSF) which triggers the proliferation of GAM via M-CSF receptor (M-CSFR) [22].

O-Vanillin demonstrated a good tolerability at even high concentrations up to 2 mM on a cellular level and 100 mg/kg (660 µM) over one month in vivo in rodents without evidence for macroscopic anomalies or cell death [11,23]. We also tested the effect of O-Vanillin on the survival of oligodendrocytes using a cell line as a model, primary cultured astrocytes and microglia, and found no impact on cell viability using the propidium iodide and Alamar Blue assays. At higher concentrations and longer exposure times, O-Vanillin decreased glioma cell survival while having no impact on the survival of glial cells. Marton et al. reported that O-Vanillin reduced the growth of a human Melanoma cell line [10]. We thus identified O-Vanillin as a potential drug to attenuate glioma growth. Since O-Vanillin was shown to penetrate the blood-brain barrier [24], there should be further studies conducted to clarify the therapeutic effect of O-Vanillin in vivo.

## 4. Materials and Methods

### 4.1. Animals

We made primary microglial cell cultures and organotypic brain slice cultures (OBSC) from C57/Bl6 wild-type mice (Charles River Laboratories, Wilmington, MA, USA) for all in vitro experiments. The mice were bred and maintained in the animal housing facility of the Max Delbrück Center as per ethical rules and approval of the local governmental institutions (01 January 2018; X9005/18; §9 and §11 of German animal protection low). The animals lived according to a 12/12 dark-light rhythm and had access to water and food ad libitum.

### 4.2. Cell Culture

Primary microglial cells were cultivated as described previously (Markovic et al., 2005) [14]. Briefly, the cerebral cortices of newborn C57Bl/6 mice were carefully cleaned of blood vessels and meninges, then the tissue was enzymatically digested with Trypsin/DNase (Gibco/Life Technologies, Carlsbad, CA, USA) and mechanically dissociated with a fire-polished glass Pasteur pipette. Cultures were incubated at 37 °C, 5% CO_2_ humidified atmosphere in complete growth medium (10% fetal calf serum (FCS)/Dulbecco´s modified Eagles Medium (DMEM with 200 mM glutamine, 100 U/mL penicillin and 100 µg/mL streptomycin all from Gibco/Life Technologies) and the next day adherent cells were washed 5 times with phosphate buffered solution (PBS)(Gibco/Life Technologies). After culturing for one week followed by culturing with L929 fibroblast cell line (RRID:CVCL_AR58; obtained from American Type Culture Collection, Manassas, VA, USA) conditioned medium for two days the microglia are now seen as floating cells or as semi-adherent cells on top of an astrocytic monolayer. These were then harvested by shaking the culture flask.

### 4.3. Astrocytes

To generate C57/Bl6 wild type astrocytes, the animals were processed as described above. After the third microglial shake off, the confluent flasks were washed thoroughly with PBS. Trypsin/EDTA was used to detach the cells. The reaction was stopped with complete growth medium and the cells were centrifuged at 500× *g* for 5 min at 4 °C. Then the cells were counted in a Neubauer chamber (Paul Marienfeld, Lauda-Koenigshoffen, Germany) according to the manufacturer´s instructions and plated as required for each experiment.

### 4.4. Glioma Cell Lines

The GL261 cells (RRID:CVCL_Y003)were obtained from the Jackson Laboratory (Bar Harbor, ME, USA). The cells were grown in DMEM with 10 % FCS, 200 mM glutamine, 100 U/mL penicillin and 100 µg/mL streptomycin (all from Gibco/Life Technologies). Red fluorescent mCherry expressing GL261 glioma cells were generated according to the protocol of Vinnakota et al. [5]. First, we transfected OmicsLinknontargeted short hairpin RNA tagged with mCherry (GeneCopoeia, Rockville, MD, USA) according to the manufacturer’s instructions. Transfected GL261 cells were then treated with 5 µg/mL puromycin (Gibco/Life Technologies) for the selection. After the 15th passage, the cell line was discarded.

#### 4.4.1. OLN93 Oligodendrocyte Cell Line

The immortalized oligodendrocyte progenitor cell line OLN93 (RRID:CVCL_5850; gift from C. Richter-Landsberg) was passaged in complete growth medium and split when confluent [25,26].

#### 4.4.2. Human Glioma Samples

All human glioma samples were provided by the Department of Neurosurgery, University Hospital Schleswig-Holstein and Department of Neurosurgery HELIOS Hospital Berlin-Buch. After the Department for Pathology confirmed the diagnosis of a malignant glioma, the cells were further processed. All patients were informed and gave their permission for further scientific processing of the tissue samples. Analysis of resected human brain tumors was performed according to the rules by the Ethical Committee of University Hospital Kiel (UKSH D447/18) and Charité (EA4/098). Briefly, tumor tissue was taken during the surgery and was placed immediately in complete growth medium for cell isolation.

All experiments were performed with Mycoplasma-free cells.

#### 4.4.3. Magnetic Cell Separation (MACS) of Human Brain TumorTissue

Glioma associated brain microglia/macrophages were isolated from the human tumor resected tissues. Fresh tissue was dissociated immediately after resection using a neural tissue dissociation kit (MiltenyiBiotec, BergischGladbach, Germany). Erythrocytes were lysed by adding 5 mL ammonium chloride solution. Thereafter, cells were resuspended in PBS containing 0.5% bovine serum albumin and 2 mM EDTA. Magnetic sorting for CD11b+ cells was then performed by using CD11b MicroBead kit (MiltenyiBiotec) following the manufacturer´s instruction. MACS into CD11b negative (Flow-through) and CD11b positive (CD11b+) enriched cell populations was done using several MACS columns in series. Both CD11b negative and CD11b+ fractions were collected. A purity check was performed after MACS by flow cytometry analysis of a small fraction of the sorted populations (BD FACS Aria, BD Biosciences, San Jose, CA, USA).

### 4.5. Production of Glioma Conditioned Medium (GCM)

GCM was prepared from the 80% confluent GL261 cultures by cultivation in complete culture medium overnight. The GCM was collected, briefly centrifuged and sterile-filtered through 0.2 µm filter mesh (Sartorius Stedim Biotech GmbH, Göttingen, Germany) and applied for further experiments.

#### Flow Cytometry

For all the flow cytometry analysis, after the treatment or isolation, cells were incubated for 30 min at 4 °C. Before fluorescence activated cell sorting (FACS), cells were stained with 2.5 µg/mL propidium iodide (Life Technologies) for 15 min. Flow cytometry was done by BD FACS Aria and data were analyzed using FlowJo software (Treestar, Ashland, OR, USA). Flow cytometry data were quantified by mean fluorescence intensity (MFI) and presented in histograms.

### 4.6. IL-6 ELISA

To investigate how the cytokine release was affected by the O-Vanillin treatment, 20,000 microglial cells were plated in a 96 well plate (Sarstedt, Nümbrecht, Germany) and maintained overnight. On the next day the cells were stimulated with Pam3CSK4 (InvivoGen, San Diego, CA, USA) and GCM for 6 h and treated with 100 µM O-Vanillin (Sigma-Aldrich, St. Louis, MO, USA). LPS (Enzo Life Sciences Inc., Farmingdale, NY, USA) was used as a positive control. After stimulation, the medium was discarded and fresh culture growth medium was added for 12 h. Then the medium was evacuated and stored at −20 °C. The IL-6 ELISA Assay was performed with ELISA Kit (R&D Systems, Minneapolis, MN, USA). For further experiments the medium was diluted 1:10 in an ELISA Buffer. Prior to the quantification of IL-6, the 96 well plates were precoated. The capture antibody was diluted in PBS according to manufacturer´s recommendations and pipetted into a 96-well microplate. The plates were sealed and incubated overnight. The next day the plates were washed 3 times with washing buffer, blocked for 1 h and washed again. Then, 100 µL of the diluted sample solution and standard solution were added to the prepared plates and incubated for 2 h at room temperature. Afterwards, the sample solutions were aspirated and the plates were washed again 3 times. The detection antibody was added and incubated for 2 h at room temperature with subsequent aspiration of the antibody and washing. After incubating the plates with 100 µL of the provided Streptavidin-HRP for 20 min and washing them again, the substrate solution was added and incubated for 20 min. To stop the reaction, we mixed 50 µL of Stop Solution into each well. The optical density of each well was analyzed using a plate reader at 450 nm (Infinite M200, Tecan, Männedorf, Switzerland).

### 4.7. Propidium Iodide (PI) Staining

A total of 20,000 neonatal C57/Bl6 microglial cells, 10,000 astrocytes and 5000 OLN93 oligodendrocytes were plated in different 96-well plates and were left to rest overnight. The next day, the cells were treated with 100 µM O-Vanillin for 24 or 48 h. Following the incubation period, the cells were washed with 200 µL PBS. Then, 100 µL complete growth medium containing 2.5 µg/mL PI (Life Technologies,) was added to stain the DNA of dead cells with permeable cell membranes. The cells were incubated for 15 min at room temperature and analyzed in a plate reader (Infinite M200, Tecan) at an excitation wavelength of 530 nm and a frequency of 25 flashes. The emission was analyzed at a wavelength of 645 nm. The fluorescence intensity of an untreated control group was defined as 100% living cells. As positive control 10 µL DMSO (Sigma-Aldrich) was added to every sample and the plate was analyzed again after 15 min. The assay was considered as working if the fluorescence intensity of incorporated PI increased at least twofold.

### 4.8. Proliferation Assay

To determine the number of proliferating cells, we analyzed the incorporation of blue fluorescent BrdU (Roche, Basel, Switzerland) into the DNA within 24 h. A total of 20,000 C57/Bl6 microglial cells were plated in a 96 well plate. Afterwards, the cells were treated with 100 µM O-Vanillin overnight. On the next morning, the medium was discarded and new complete growth medium containing fluorescent labeled BrdU and 100 µM O-Vanillin was added. The cells were incubated for another 24 h.

As a negative control we used dead cells after incubation in 10% DMSO for 24 h. As a positive control we used the well-known inductor of microglia proliferation, mMSCF (PeproTech, Rocky Hill, CT, USA) [27]. The cells were analyzed in a plate reader (Infinite M200, Tecan). While mMCSF induced a significant increase in proliferation, the DMSO incubated cells showed no evidence for BrdU incorporation.

### 4.9. Alamar Blue Assay

The metabolism assay Alamar Blue (Alamar Blue Cell Viability Reagent, Thermo Fisher Scientific, Waltham, MA, USA) was applied for both primary murine neonatal microglia and murine GL 261 cells. Primary murine neonatal microglia were used directly after preparation from newborn C57BL/6 wildtype mice. Murine glioma GL261 cells were harvested from a 25 cm^2^ cell culture flask (Sarstedt). The cell number was adjusted to 10,000 cells/mL. Next, the cells were treated with different O-Vanillin concentrations and incubated for 3, 6 and 24 h. Fresh complete growth medium was added to the control wells. Finally, all media were replaced by 100 µL of fresh 10% Alamar Blue solution made by diluting Alamar Blue dye to fresh medium and the cells were incubated for further 3 h at 37 °C and 5% CO_2_. The Alamar Blue absorption spectrum in cells was measured using a plate reader (Infinite M200). The data was analysed according to the manufacturer´s protocol (Thermo Fisher Scientific, AlamarBlue Cell Viability Reagent).

### 4.10. RNA Isolation/qPCR

For all RNA dependent experiments up to 300,000 cells were plated in a 12-well plate (Sarstedt). The standard treatment regimen was 100 µM O-Vanillin for 6 h. Isolated human CD11B positive cells were treated for 12 h. Cells were washed in cold PBS (Life technologies) to remove redundant complete growth medium. The isolation was carried out using a Promega RNA Isolation Kit (ReliaPrep RNA Cell Miniprep System, Promega, Fitchburg, MA, USA) following manufacturer´s instructions. The isolated RNA was dissolved in 15 µLRNAse free water (Sigma-Aldrich) and stored at −20 °C. The quality and total amount of RNA was analyzed using a Nano Drop (NanoDrop8000 Spectrophotometer, Thermo Fisher Scientific, Waltham, MA, USA). RNA was transcribed into cDNA using a TaKaRa cDNA Synthesis Kit (Takara Bio INC., Kyoto, Japan) following manufacturer´s instructions. The cDNA was stored at −20 °C. qPCR was performed in 96 well real time pPCR fast plates (7500 Fast Real-Time PCR System, Life Technologies). A master mix containing 10 µL SYBR Green (Life Technologies), 8 µLRNAse free water and 1 ng of cDNA was added to each well. The forward and reverse primers (Table S1) were added at a concentration of 10 pg/mL. Then the plate was covered with an adhesive film (Applied Biosystems, Thermo Fisher Scientific) and centrifuged at 1000 rcf for 1 min at 4 °C (Centrifuge 5417 R Eppendorf, Wesseling-Berzdorf, Germany). TBP (Tata box binding protein) was used as house-keeping gene [28]. We provide the raw qPCR values in data suppliment.

### 4.11. Organotypic Brain Slices

All working steps were carried out after all used material was disinfected with 70% ethylalcohol (EtOH) for at least 30 min. The brain slices were obtained from 13- to 16-day-old C57/Bl6 wild type mice. The mice were sacrificed by cervical dislocation and decapitated, brains were carefully removed and immediately transferred to ice cold PBS. The cerebellum and olfactory bulbs were cut off. The brain was glued (UHU Sekundenkleber, UHU GmbH and Co. KG, Bühl, Germany) in an upward position with its caudal end to the cutting table of the vibratome (MicrotomHM 650 V, Thermo Scientific) and its ventral end to an already fixed 5% Agar-Agar block (KobeI, Roth, Karlsruhe, Germany). The cutting table was fixed in a cooled cutting chamber and covered with ice cold PBS. A vibratome was used to cut the brain into 250 µm slices. The slices were directly transferred with a glass pipette to ice cold Hanks’ Balanced Salt Solution (HBSS) (Thermo Fisher Scientific). Up to 3 brain slices were plated on one semi-permeable membrane insert (Falcon cell culture inserts, 0.4 µm pore size, Corning Life Sciences, Amsterdam, The Netherlands) in a 6-well plate filled with 1 mL complete growth medium. Red fluorescent GL261 glioma cells were grown confluent and harvested. The cells were further concentrated up to 10,000/µL and 1 µL drawn up into a Hamilton 1 µL precision Syringe (Microliter Syringe Model Model 701 RN SYR, Hamiltion Company, Bonaduz, Switzerland) with micro needle. The syringe was attached to a customized micromanipulator. Twenty-four hours after preparing the murine brain slices, the culture inserts were transferred to a working 6-well plate equipped with a micromanipulator. All working steps were performed at 37 °C in complete growth medium. The precision syringe was adjusted 250 µm above the culture insert membrane and the brain slices were positioned. Then, 10,000 mCherry GL261 cells were injected into the region of basal ganglia in a depth of 150 µm by slowly pushing the plunger. During injection the syringe was slowly moved upwards. The mCherry GL261 cells were injected over a range of 50 µm. After injections of red fluorescent GL261, the culture inserts were transferred to a new plate filled with culture medium containing different concentrations of O-Vanillin. The brain slices were incubated for 5 days. The medium was renewed every two days.

### 4.12. Fixation of Brain Slices

Brain slices were fixed with 4% paraformaldehyde (Thermo Fisher Scientific, Paraformaldehyde, Methanol-free) for 1 h and the cell nuclei were stained with blue fluorescent dye Hoechst (Thermo Fisher Scientific, Hoechst 33,342 Solution) for 30 min at room temperature. After the brain slices were carefully cut out from the insert membrane and transferred on microscopy slides, mounted with Aqua-Poly/Mount (Polysciences, Inc., Aqua-Poly/Mount) and covered with coverslip.

### 4.13. Microscopy

All micrographs were taken using a Zeiss LSM 710 confocal microscope (Carl Zeiss, Oberkochen, Germany) with a 20× objective. Injected mCherry GL261 gliomas were visualized by excitation wavelength 550 nm and emission wavelength 645 nm and further analyzed at the depth of maximal surface area.

### 4.14. Tumor Size Measurements

The tumour area and volume were calculated using Imaris 8 software (Version 9.5.0, Bitplane Inc., St. Paul, MN, USA). Tumor volumes of high-resolution Zeiss LSM 710 confocal microscopy stacks were 3D rendered by application of 1 µm object detail, 15 threshold background and 1000 tridimensional pixels (voxels). The surface objects obtained were unified in one single object, and volume mean values were extracted.

### 4.15. Statistical Analysis

All analyses were performed using Prism Graphpad (Version 8.4.1, GraphPad Software, San Diego, CA, USA) and Microsoft Excel 2016. Statistically significant differences were determined with the Student´s *t*-test for parametric testing. One-way ANOVA was used to compare multiple groups with Bonferroni post-hoc test. Significance was defined at *p* values < 0.05 (*), <0.01 (**) and <0.001 (***).

## Supplementary Materials

The following are available online at https://www.mdpi.com/1422-0067/21/8/2959/s1. Figure S1: Treatment with O-Vanillin does not affect the cell death of microglial cells; Table S1: List of primers. Supplimental material 1: raw qPCR data.

## Abbreviations

MMP	matrix metalloproteinases
TLR	Toll-like receptor
GAM	glioma associated microglia and macrophages
IL	interleukin
iNOS	inducible nitric oxide synthase
TCGA	the cancer genome atlas
OBSC	organotypic brain slice culture
GCM	glioma conditioned medium
