# Supplementary material for: O-Vanillin Attenuates the TLR2 Mediated Tumor-Promoting Phenotype of Microglia
Source: Int J Mol Sci. 2020 Apr 22;21(8):2959. doi: 10.3390/ijms21082959 (PMC7215774; doi:10.3390/ijms21082959)
Supplement: Supplementary file 1 [file ijms-21-02959-s001.zip › Supl. Table 1.docx]

*Mouse Primers*

| **Target** | **Forward** | **reverse** |
| --- | --- | --- |
| *beta-Actin* | CGT GGG CCG CCC TAG GCA CCA | CTT AGG GTT CAG GGG GGC |
| *inos* | TCACGCTTGGGTCTTGTTCA | TGAAGAGAAACTTCCAGGGGC |
| *mmp14* | GTG CCC TAT GCC TAC ATC CG | CAG CCA CCA AGA AGA TGT CA |
| *mmp9* | CATTCGCGTGGATAAGGAGT | ACCTGGTTCACCTCATGGTC |
| *tbp* | AAGGGAGAATCATGGACCAG | CCGTAAGGCATCATTGGACT |

*Human Primers*

| **Target** | **Forward** | **reverse** |
| --- | --- | --- |
| *GAPDH* | GTC AGT GGT GGA CCT GAC CT | AGG GGA GAT TCA GTG TGG TG |
| *MMP14* | CGCTACGCCATCCAGGGTCTCAAA | CGGTCATCATCGGGCAGCACAAAA |
| *MMP9* | AAGGCGCAGATGGTGGAT | TCAACTCACTCCGGGAACTC |
| *TLR2* | TCTCCCATTTCCGTCTTTTT | GGTCTTGGTGTTCATTATCTTC |
