# Supplementary material for: O-Vanillin Attenuates the TLR2 Mediated Tumor-Promoting Phenotype of Microglia
Source: Int J Mol Sci. 2020 Apr 22;21(8):2959. doi: 10.3390/ijms21082959 (PMC7215774; doi:10.3390/ijms21082959)
Supplement: Supplementary file 1 [file ijms-21-02959-s001.zip › Supplimental material 1.pdf]

Human CD11B pos. Cells

Confirmed GBM
500 000 MACS sorted cells per well
Ctrl Group vs 100µM O-Vanillin
Overnight treatment

Tumor
OP20012017
OP25012017

Sample	Target	CT Mean	GAPDH CT	Difference	Corrected	Fold Change
OP250117Ctrl1	MMP14	33.31617737	28.7523861	0.04691633	33.269261	1
OP250117Ctrl2	MMP14	33.26809692	29.1502399	0.44477018	32.8233267	1
OP250117OV1	MMP14	33.33500671	28.6831036	-0.02236621	33.3573729	0.7341566
OP250117OV2	MMP14	34.2047081	28.8599758	0.15450605	34.0502021	0.4272418
OP200117Ctrl	MMP14	34.63786697	29.7565556	0.34374428	34.2941227	1
OP200117OV	MMP14	34.2454567	29.069067	-0.34374428	34.589201	0.81502811
H2O	MMP14		Undetermined			
		Average 200117	28.7054698			
		Average250117	29.4128113			

Sample	Target	CT Mean	GAPDH CT	Difference	Corrected	Fold Change
OP250117Ctrl1	MMP9	25.60007286	28.7523861	0.04691633	25.5531565	1
OP250117Ctrl2	MMP9	25.75378036	29.1502399	0.44477018	25.3090102	1
OP250117OV1	MMP9	26.15553856	28.6831036	-0.02236621	26.1779048	0.64853295
OP250117OV2	MMP9	27.18517685	28.8599758	0.15450605	27.0306708	0.30319952
OP200117Ctrl	MMP9	29.5340271	29.7565556	0.34374428	29.1902828	1
OP200117OV	MMP9	29.95483208	29.069067	-0.34374428	30.2985764	0.46384235
H2O	MMP9		Undetermined			
		Average 200117	28.7054698			
		Average250117	29.4128113			

Tumor
OP07032017

Sample	Target	CT	GAPDH CT	Difference	Corrected	Fold Change
Ctrl1	MMP14	23.34068108	20.3786182	0.40108824	22.9395928	1
Ctrl2	MMP14	23.21336746	19.8087578	-0.16877222	23.3821397	1
OV1	MMP14	23.25008392	20.0320873	0.05455732	23.1955266	0.83744494
OV2	MMP14	23.11669731	19.6906567	-0.28687334	23.4035707	0.98525497
		Average	19.97753			

Sample	Target	CT	GAPDH CT	Difference	Corrected	Fold Change
Ctrl1	MMP9	17.92854118	20.3786182	0.40108824	17.5274529	1
Ctrl2	MMP9	17.50784302	19.8087578	-0.16877222	17.6766152	1
OV1	MMP9	18.5588398	20.0320873	0.05455732	18.5042825	0.50809511
OV2	MMP9	17.16085434	19.6906567	-0.28687334	17.4477277	1.17193094
		Average	19.97753			

Tumor
OP28022017 (average of duplicates)

Sample	Target	Mean CT	GAPDH CT	Difference	Corrected	Fold Change
Ctrl	MMP14	22.69521713	24.9339294	0.08513641	22.6100807	1
100µM OV	MMP14	23.17624664	24.7636566	-0.08513641	23.2613831	0.63670529
H2O	MMP14					
		Average	24.848793			

Sample	Target	Mean CT	GAPDH CT	Difference	Corrected	Fold Change
Ctrl	MMP9	23.25020218	24.9339294	0.08513641	23.1650658	1
100µM OV	MMP9	24.57823944	24.7636566	-0.08513641	24.6633759	0.35396777
H2O	MMP9					
		Average	24.848793			

Mean CT Values were corrected using the GAPDH Average of each qPCR
We calculated the relative values of the qPCR log function defining the ctrl as 2^(Ctrl-Ctrl)
and the Fold change as 2^(Ctrl-Treated)
