# Supplementary figures and images for: O-Vanillin Attenuates the TLR2 Mediated Tumor-Promoting Phenotype of Microglia
Source: Int J Mol Sci. 2020 Apr 22;21(8):2959. doi: 10.3390/ijms21082959 (PMC7215774; doi:10.3390/ijms21082959)

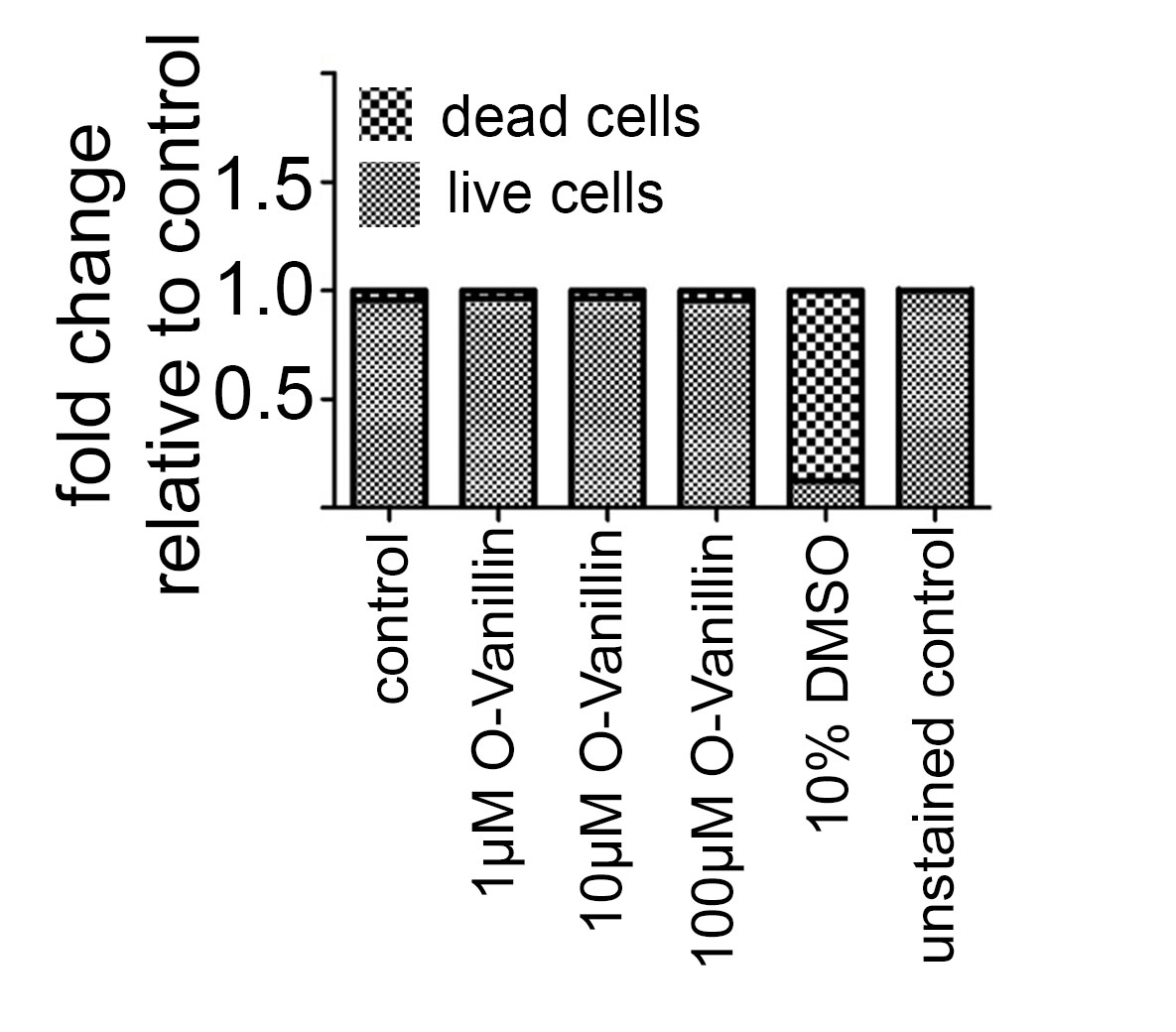

Supplement: Supplementary file 1 [file ijms-21-02959-s001.zip › Supl Fig1.jpg]
